# Potential for Integrating Mental Health Specialist Video Consultations in Office-Based Routine Primary Care: Cross-Sectional Qualitative Study Among Family Physicians

**DOI:** 10.2196/13382

**Published:** 2019-08-19

**Authors:** Mariell Hoffmann, Mechthild Hartmann, Michel Wensing, Hans-Christoph Friederich, Markus W Haun

**Affiliations:** 1 Department of General Internal Medicine and Psychosomatics Heidelberg University Heidelberg Germany; 2 Department of General Practice and Health Services Research Heidelberg University Heidelberg Germany

**Keywords:** video consultations, videoconferencing, integrated behavioral health, primary care, health services research, mental health

## Abstract

**Background:**

Although real-time mental health specialist video consultations have been proposed as an effective care model for treating patients with mental health conditions in primary care, little is known about their integration into routine practice from the perspective of family physicians.

**Objective:**

This study aimed to determine the degree to which family physicians advocate that mental health specialist video consultations can be integrated into routine primary care, where most patients with mental health conditions receive treatment.

**Methods:**

In a cross-sectional qualitative study, we conducted 4 semistructured focus groups and 3 telephonic interviews in a sample of 19 family physicians from urban and rural districts. We conducted a qualitative content analysis applying the Tailored Implementation in Chronic Diseases framework in a combined bottom-up (data-driven) and top-down strategy for deriving key domains.

**Results:**

Family physicians indicated that mental health specialist video consultations are a promising and practical way to address the most pressing challenges in current practice, that is, to increase the accessibility and co-ordination of specialized care. Individual health professional factors were the most frequently discussed topics. Specifically, family physicians valued the anticipated clinical outcomes for patients and the anticipated resources set for the primary care practice as major facilitators (16/19, 84%). However, family physicians raised a concern regarding a lack of facial expressions and physical interaction (19/19, 100%), especially in emergency situations. Therefore, most family physicians considered a viable emergency plan for mental health specialist video consultations that clearly delineates the responsibilities and tasks of both family physicians and mental health specialists to be essential (11/19, 58%). Social, political, and legal factors, as well as guideline factors, were hardly discussed as prerequisites for individual family physicians to integrate mental health specialist video consultations into routine care. To facilitate the implementation of future mental health specialist video consultation models, we compiled a checklist of recommendations that covers (1) buy-in from practices (eg, emphasizing logistical and psychological relief for the practice), (2) the engagement of patients (eg, establishing a trusted patient-provider relationship), (3) the setup and conduct of consultations (eg, reliable emergency plans), and (4) the fostering of collaboration between family physicians and mental health specialists (eg, kick-off meetings to build trust).

**Conclusions:**

By leveraging the primary care practice as a familiar environment for patients, mental health specialist video consultations provide timely specialist support and potentially lead to benefits for patients and more efficient processes of care. Integration should account for the determinants of practice as described by the family physicians.

**Trial Registration:**

German Clinical Trials Register DRKS00012487; https://www.drks.de/drks_web/navigate.do? navigationId=trial.HTML&TRIAL_ID=DRKS00012487

## Introduction

### Background

For decades, primary care has been the de facto mental health care outpatient system in most countries. In recent years, the management of mental health conditions in primary care has further increased because of the higher prevalence and regional shortages of mental health care providers [1]. Collaborative mental health care integrating mental health specialists in primary care practices has been proposed as a possible solution. However, its coordination with primary care has been found to be a persistent challenge [2,3]. To address this challenge, clinicians and policy makers have recently proposed real-time mental health specialist video consultations as a service delivery model [4-6]. Telepsychiatry technologies such as mental health specialist video consultations are integrated, patient-centered care models that can be classified into low-, moderate-, and high-intensity models. These levels depend on the complexity of the intervention as well as the amount of resources, such as the extent to which the primary care provider or specialist is involved [7]. In this study, we examined the potential for integrating mental health specialist video consultations as a moderate-intensity model into office-based routine primary care. Within our model, family physicians referred patients presenting in primary care with depression and/or anxiety to a mental health specialist. The mental health specialist will then schedule and conduct the mental health specialist video consultation with the patients in the primary care practice. The mental health specialist intervention is time limited and aimed at diagnostics, care planning, and crisis management or brief psychotherapy. To date, there have been some pioneering studies indicating that video consultations work well for health care settings [8-12]. Specifically, in randomized controlled trials that were conducted within the unique context of the Veterans Health Administration system and federally qualified health centers, video consultations were effective for depression [8,9]. Furthermore, commercial telepsychiatry providers have established nonclinical physician extenders as virtual care navigators for referring patients from primary care to a telepsychiatrist [10]. There is also preliminary evidence that video consultation–based collaborative care decreases costs and improves access to mental health care, especially in rural areas, by reducing travel time [11,12].

### Implementation of Mental Health Specialist Video Consultations Into Primary Care

Although these findings are promising, we do not know whether and to what extent practicing family physicians themselves are convinced that mental health specialist video consultations can be integrated into routine practice. Moreover, although Powell et al recently reported that primary care patients are quite willing to engage in video consultations, the prerequisites for the amount of time needed for their adoption from the family physicians’ perspective are unknown [13,14]. However, for the development of successful implementation strategies for routine care, it is crucial to allow family physicians, who are the central stakeholders in routine care, to evaluate the anticipated benefit of the proposed models. Therefore, the purpose of this qualitative focus group study was to assess the potential for the integration of mental health specialist video consultations into office-based routine primary care based on the analysis of family physicians’ perceptions of current practice.

## Methods

### Study Design and Conceptual Framework

We conducted a cross-sectional qualitative study with semistructured focus groups as a naturalistic data collection method to assess the collective sense making of family physicians and to explore experiences and perceptions through the interaction of the participants [15]. We opted for focus groups, given that they are more appropriate than interviews when people do not have a personal stake in the topic [16] and that focus group participants usually have less constrained discussions than those in individual interviews [17]. We conducted telephonic interviews with family physicians who were not able to participate in a focus group for organizational reasons. By integrating focus groups and interviews, we were able to (1) avoid the withdrawal of family physicians who were particularly interested in study participation and (2) capture complementary perspectives on mental health specialist video consultations [18] and therefore gather broader information. To capture both current real-world practice and the potential integration of video consultations, we followed a pragmatic approach based on the critical realist position. This allowed us to derive recommendations for future interventional studies on mental health specialist video consultations [19]. This study was approved by the ethics committee of the medical faculty at Heidelberg University (S-197/2017) and preregistered with the German Clinical Trials Register (DRKS00012487). We followed the COnsolidated criteria for REporting Qualitative research guidelines for reporting qualitative study results [20].

### Participants and Recruitment

We applied purposeful sampling to account for a diverse range of participants and information [21]. Specifically, in an initial survey study (results not presented here) on the integration of mental health specialist video consultations, we invited all 788 family physicians registered with the Association of Statutory Health Insurance Physicians in 1 urban and 4 rural districts (from a total of 35 districts in Baden-Wuerttemberg, 1 of 16 German federal states) to participate in a focus group. Apart from registration, there were no other eligibility criteria for family physicians. Overall, 107 family physicians (13.6%, 107/788) responded to the survey, 41 of whom showed interest in participating in a focus group. We conducted phone calls with 35 family physicians to schedule a focus group appointment with a maximum of 6 participants each. One family physician took part in a focus group without a formal invitation but rather following another participating family physician’s suggestion. Overall, 16 family physicians refused to participate, most frequently because of holiday leave (n=4) and lack of interest (n=4). In addition, 7 family physicians were not contacted because of earlier-than-expected data saturation. The latter was achieved when no additional insights emerged from data, and the content began to repeat. Overall, we conducted 4 focus groups (range: 2-6 participants, 90-120 min) involving 16 family physicians at Heidelberg University Hospital alongside individual telephonic interviews (40-55 min) with 3 family physicians. We offered a nonadvertised individual monetary compensation of €50.

### Data Collection

We developed a semistructured question guide to prompt group discussions and interviews (Multimedia Appendix 1). The questions focused on how family physicians perceived current health care for patients with mental disorders, the potential for integrating mental health specialist video consultations into office-based routine primary care, and the determinants of the implementation of mental health specialist video consultations. We piloted the guide on 1 family physician and 1 senior health services researcher. It was also reviewed after the first focus group. After obtaining written informed consent from all participants, the first author (Doctor of Philosophy student, sociologist, and expertise in qualitative research) and the last author (Doctor of Medicine, internal medicine specialist, senior researcher, and content expert for mental health services) moderated the focus groups. To stimulate the discussion, the moderators presented a 7-min video clip illustrating the mental health specialist video consultation model. The interviews were conducted by the first author who verbally described the model. All focus groups and interviews were audio-recorded and uploaded to a secure server of Heidelberg University Hospital, which was only accessible to the research team. We analyzed the participants’ sociodemographic and primary care practice data that were collected in the initial mail survey.

### Data Analysis

First, before anonymizing the data, a professional transcription service conducted verbatim audio transcriptions of the recordings. Second, 2 authors (MH and MWH) independently conducted a qualitative content analysis of the first focus group in MAXQDA 12 (VERBI Software). To develop the code system, we followed a combined bottom-up (data-driven) and top-down strategy [22]. For the latter, we applied the 7 domains of the Tailored Implementation in Chronic Diseases (TICD) framework as an analytical lens to identify the determinants of practice [23]. The TICD accounts for multiple levels (micro- and macrolevels) and stakeholder perspectives (eg, patients and health care providers). Third, both researchers compared their code systems and resolved disagreements in a final version. Fourth, we applied the code system to the remaining transcripts. To ensure representation of all key aspects, we modified the codes when new aspects emerged. We summarize the key domains, including definitions and supporting quotes in Multimedia Appendix 2.

## Results

### Sample

Table 1 shows the sociodemographic characteristics of the 19 participating family physicians.

**Table 1:** Sample description (N=19).

Variable		Value
Female sex, n (%)		9 (48)
Age (years), mean (SD)		57.5 (7.3)
Years in office-based practice, mean (SD)		18.3 (9.7)
**Type of practice, n (%)**		
	Solo practice	12 (63)
	Shared practice	6 (32)
	Group practice	1 (5)
**Areas of recruitment, n (%)**		
	Cities (densely populated areas)	2 (11)
	Towns and suburbs (intermediate density areas)	14 (74)
	Rural areas (thinly populated areas)	3 (16)
Additional qualification in psychotherapy, n (%)^a^		4 (21)
**Average number of patients per quarter, n (%)**		
	<500	1 (5)
	501-1000	6 (32)
	1001-1500	6 (32)
	>1500	6 (32)
**Patients with mental health conditions per week, n (%)**		
	1-5	1 (5)
	5-10	3 (16)
	10-15	7 (37)
	>10-15	8 (42)

^a^Multiple responses possible.

### Depiction of Current Practice

For family physicians, the coordination of care for patients presenting with mental health disorders was a persistent challenge in current practice. Waiting times and an insufficient number of available mental health specialists (14/19), as well as a lack of professional exchanges between family physicians and mental health specialists (12/19), were major difficulties:

It is very difficult because we have such poor access to psychotherapists. [...] They [the patients] must wait for a quarter of a year to half a year to get an appointment.

Interview 3

These barriers consistently produced a precarious psychological dilemma. On the one hand, family physicians did not have enough resources (eg, because of time restrictions) to provide mental health care themselves for all patients in need (12/19):

Psychotherapy cannot be integrated into everyday primary care practice. However, the first step to getting a feeling or becoming pretty sure that this patient would need to be referred to someone, to a psychotherapist...this takes more than the usual ten- or fifteen-minutes during the consultation...Um...to open Pandora’s box, that takes more time. It...um...the patient must feel that he can open up himself. That is quite a time challenge because during flu season, I must admit, that I just don’t listen, I just don’t go there. That is not possible then.

Focus group 1

Family physicians could not refer patients to mental health specialists because of unavailability. On the other hand, observing that patients remained undertreated was at odds with the professional ethics of the family physicians, who generally commit themselves to providing comprehensive care to their patients (6/19, 32%):

Interviewer: How do you handle the less urgent cases?

Bm1: Um, those are, um, mostly unserved, or I improvise and um, I am not so happy with that.

Focus group 3

### Determinants for Implementing Mental Health Specialist Video Consultations

Family physicians perceived mental health specialist video consultations as a potentially effective approach to address the gaps in current practice. Specifically, family physicians felt that mental health specialist video consultations would increase low-threshold access and improve coordination to provide mental health care in primary care. In our analysis, we identified 44 subdomains and grouped them into the 7 domains of the TICD framework: individual health professional factors; patient factors; professional interactions; incentives and resources; capacity for organizational change; social, political and legal factors; and guideline factors. In the following sections, we present our results along these 7 domains arranged in descending order by code frequency. We report code frequency in brackets behind each code to provide information about data saturation and complement our findings. Two domains, namely, social, political, and legal factors and guideline factors, were hardly discussed and are not reported.

### Individual Health Professional Factors

Family physicians’ perceptions of the integration of mental health specialist video consultations into primary care were strongly influenced by both the anticipated clinical outcomes and the anticipated resources made available for primary care practice. Specifically, family physicians considered mental health specialist video consultations to potentially facilitate seamless and low-threshold access to specialized mental health care (7/19, 37%):

However, I do see an advantage in having a low-threshold option, to refer a patient to a consultation with a professional, without having to enlist him somewhere, without having him to drive anywhere and so on.

Focus group 2

By enabling timely referrals, family physicians argued, mental health specialist video consultations would lead to relatively rapid clinical improvement in patients, which emerged as the foremost purpose of family physicians’ professional identity:

I could offer something to my patients, not for the purpose of advertisement, but I could actually propose something that makes patients feel better. And that’s why I attend to the patient in the first place.

Focus group 4

Moreover, several family physicians expected that, to some extent, mental health specialist video consultations would make resources available for the entire primary care team and, in the long term, lead to more efficient daily workflows (9/19, 47%). They underscored that the major benefit would entail psychological relief for them as they would now be able to trust that the adequate treatment has been initiated:

They [mental health specialist video consultations] may produce some psychological relief by letting me know that I did something good for the patient [...] Of course, the feeling that I did something meaningful for the patient would be relieving.

Focus group 4

Furthermore, considering patient outcomes, some family physicians underscored the difference between virtual and face-to-face consultations (6/19, 32%). Some family physicians stated that with certain patient groups, there may be a risk of not being able to establish a sufficiently stable therapeutic relationship through mental health specialist video consultations. Consequently, family physicians argued that the establishment and monitoring of a solid patient-provider relationship should be the top priority when implementing mental health specialist video consultations. Otherwise, mental health specialist video consultations might alienate patients from care providers. One particularly concerned family physician stated the following:

Well, as I said, I think they [mental health specialist video consultations] may work with some people, but in principle, it is different from sitting across from someone. There you get information you won’t get through a screen.

Focus group 2

Specifically, family physicians worried that nonverbal communication may be constricted in mental health specialist video consultations and that this anticipated limitation would become particularly apparent in emergency situations:

So, is the therapist actually able to assess someone through the camera in an empathetic manner? What happens if one raises a particular issue and he [the patient] leaves the room? [...] If he screams and pulls the cables out

Focus group 1

In every focus group and interview, family physicians noted the importance of facial expressions, gestures, and even physical interaction in such situations and advocated for technical solutions optimally supporting nonverbal communication. In addition, most family physicians considered a viable contingency or emergency plan for mental health specialist video consultations, clearly delineating responsibilities and tasks for both family physicians and mental health specialists as essential (11/19, 58%). For instance, they mentioned collaborative debriefing between the family physician and the mental health specialist as a consultant or expert after a mental health video consultation as well as hotline support for patients as potential solutions:

Yes, I would welcome, um, an interdisciplinary debriefing or a time window for resolving open questions.

Focus group 1

At least he [the patient] has to have some sort of hotline number [to call]. If he still had problems, and if his primary care physician and office were closed, he would still have a contact person to turn to. Something like this perhaps.

Focus group 4

### Patient Factors

Family physicians discussed several patient-related themes, namely, target groups for mental health specialist video consultations, potential preferences, and barriers for technology-based interventions such as mental health specialist video consultations that patients might anticipate.

First, family physicians regarded certain environmental conditions as prerequisites for fostering patient involvement (12/19, 63%). For instance, family physicians wanted to have a designated room available to ensure confidential consultations. Second, family physicians considered mental health specialist video consultations to be generally suitable for patients but were ambivalent concerning certain groups. A few family physicians expected the elderly to be more hesitant in accepting professional psychosocial support in general (5/19, 26%). Family physicians suspected that elderly patients are less familiar with computer technology and therefore more likely to refuse video consultations:

Many older people state that they do not want to see a psychotherapist; these people consequently won’t go for a video consultation either.

Interview 1

Nevertheless, some family physicians regarded elderly patients as potentially open-minded toward and interested in mental health specialist video consultations (6/19, 32%). To this end, 1 family physician gave an illustrative example:

I am always surprised to see my 90-year-old grandmas who use Skype without any difficulties. They have just acquired the necessary skills. From my perspective, the barrier to adopt the technology is not large at all.

Focus group 1

To facilitate patient engagement, family physicians felt responsible for introducing the patient to the mental health specialist as part of the first session and for being available to follow up with the patient beyond the mental health specialist video consultation. From the family physicians’ perspectives, patients (1) who had physical-mental health comorbidities or medically unexplained symptoms (eg, unspecific gastrointestinal complaints; 4/19, 21%), (2) who hesitated to seek mental health care, (eg, because of stigma; 5/19), and (3) who were immobile, particularly in rural areas (4/19, 21%), would most likely benefit from mental health specialist video consultations. Some family physicians considered it necessary to explicitly encourage patients to try video consultations and highlighted that each patient would have to continuously consult with the same mental health specialist (5/19, 26%).

### Professional Interactions

Family physicians eagerly discussed collaborative aspects and responsibilities related to mental health specialist video consultations. First, they highly appreciated the possibility of collaborating with the mental health specialists (10/19, 53%). Specifically, family physicians regarded brief case discussions with mental health specialists via colleague-to-colleague video calls as an opportunity to validate or revisit their initial diagnostic assessment and allow for treatments to be tailored according to patients’ needs and social environments:

I think that they [mental health specialist video consultations] support you in your work as a family physician. After the consultation, it can be discussed what has been done and whether there remains anything urgent to manage or clarify

Focus group 2

Second, concerning the distribution of tasks among the involved health care personnel, according to family physicians, medical assistants would have to be responsible for administrative and organizational tasks (eg, appointment allocation and follow-up calls; 10/19, 53%). The family physicians themselves would be responsible for referring patients to the mental health specialist video consultations, whereas family physicians described the role of mental health specialists mainly as consultants with secondary care expertise. To foster a good relationship with the mental health specialists, family physicians demanded that there would be an initial kick-off meeting to meet each other in person.

### Incentives and Resources

First and most importantly, most participants stated that reimbursement for mental health specialist video consultations should at least cover their costs for the use of the room where mental health specialist video consultations would be conducted and for additional personnel resources (13/19, 68%). The latter refers to family physicians’ statements that family physicians or their medical staff might carry out some tasks related to the intervention, such as initializing the mental health specialist video consultations via the Web platform. Second, family physicians considered the unknown amount of spatial resources (eg, a room in the family physicians’ practice to provide a confidential environment for conducting mental health specialist video consultations), personnel, and time resources initially necessary for the integration of mental health specialist video consultations into their practice as a main barrier because of the already tightly organized day-to-day routine. For instance, family physicians wanted themselves or their medical assistants to be responsible for initializing the individual mental health specialist video consultation via the Web platform. Therefore, the setup and implementation of mental health specialist video consultations should account for existing structures and workflows in the given practice. For instance, family physicians stated that the mental health specialist video consultations should be conducted outside the usual consultation hours so that a designated room can be guaranteed. Fixed time slots were expected to facilitate the integration. Third, with respect to technology, family physicians named several basic requirements: stable network connectivity, high visual definition, minimized speech delay, and instant technical service support, alongside training sessions for both practice staff and mental health specialists.

### Capacity for Organizational Change

Family physicians rarely addressed this aspect spontaneously. Obviously, the anticipated capacity for change in practices was linked to the intrinsic motivation of the individual family physician. However, family physicians emphasized that the prospect of workload relief resulting from the intervention might foster readiness for change within the medical profession (2/19, 10.5%). One family physician also suspected that the family physician’s age might determine his or her intent to adopt technology-based interventions with digital natives assumed to be more open minded (1/19, 5.3%):

It has something to do with being curious. And I can imagine that younger colleagues may be even more curious.

Focus group 2

### Distinctions

First, a comparison of data from cities (2/19, 10.5%), towns or suburbs (14/19, 74%), and rural areas (3/19, 16%) did not reveal any major distinctions. Second, a comparison between solo (12/19, 63%) and group/shared practices (7/19, 37%) indicated that participants from group or shared practices slightly less frequently discussed help for patients or relief for family physicians as expected benefits or outcomes of the intervention. In fact, 5 of 7 participants from group or shared practices valued help for patients or relief for family physicians as potential outcomes, whereas 12 of 12 participants from solo practices expected 1 of the 2 potential outcomes.

## Discussion

### Principal Findings

In this study, we investigated the potential for integrating real-time mental health specialist video consultations in primary care among family physicians working in urban and rural practices. Family physicians perceive current practice as fragmentary and deficient. From their perspective, mental health specialist video consultations are a promising and practical way to address the most pressing gaps, that is, to increase the access to and coordination of specialized care. With respect to the implementability of mental health specialist video consultations in primary care, we were able to derive specific recommendations that cover (1) buy-in from practices (eg, emphasizing potential logistical and psychological relief for the practice), (2) the involvement of patients (eg, establishing and securing a trusted patient-provider relationship), (3) the setup and conduct of consultations (eg, solid emergency plans in place), and (4) the fostering of collaboration between family physicians and mental health specialists (eg, in person kick-off meetings to build trust). With respect to both future video consultation applications in routine care as well as feasibility and full-scale intervention trials, we have summarized these recommendations in a checklist that supports stakeholders in accounting for determinants of implementation (Multimedia Appendix 3).

Previous work on video consultations has been limited to efficacy trials and postimplementation studies on perceptions, acceptance, and satisfaction [8,9,13,24-26]. In 2 efficacy trials, Fortney et al found video consultations to be a promising mode of delivery of mental health care [8,9]. However, both trials were in unique contexts (ie, the Veterans Health Administration system and rural federally qualified health centers) and did not apply any participatory assessments to capture the perspective of the professionals and patients. Studies assessing the determinants of the integration of technology-based mental health care models into primary care prospectively have been missing. To the best of our knowledge, our study is the first to provide in-depth qualitative findings on the anticipated benefit of video consultations for patients with mental health conditions in office-based primary care. In the following paragraphs, we therefore discuss our results against the background of more general findings on applying video consultations to tackle medical problems. First, from the patients’ perspective, video consultations are welcomed in settings as varied as primary care [13], emergency medicine, and radiology [24]. Concerns about the practicability of video consultations have rarely been explored and have therefore remained rather unspecific [13]. From the perspective of family physicians, we were able to characterize more specific challenges, such as handling emergency situations virtually. We were also able to address potentially sustainable solutions for these challenges with family physicians who were very familiar with routine care conditions. Second, from the health care personnel’s perspective, staff and financial resources constitute the main barriers for the integration of Web-based interventions in general [27] and video consultations in particular [25,28]. Our work adds that, in addition to these organizational factors, family physicians focus on establishing a reliable therapeutic relationship in the mental health specialist video consultation setting. The latter and patient satisfaction are substantial prerequisites for the effectiveness and acceptance of telemental health models such as mental health specialist video consultations for patients as well as health care professionals [26]. Hence, family physicians, as persons of trust, could support particularly skeptical patients in trying out mental health specialist video consultations, and family physicians could perform *warm handoffs* to refer patients by means of a personal introduction [29]. At this point, the unique advantage of embedding mental health specialist video consultations directly into primary care practice, an environment with which patients often have been familiar for decades, becomes apparent in our study. Only 1 study focused on collaborative aspects from the perspective of health care personnel using video consultations in primary care [30]. The main result, namely, that staff mostly want to consult with specialists in cases of diagnostic uncertainty, is in accordance with our findings. However, we also found that collaboration should be based on an initial personal encounter between family physicians and specialists. Similar to previous investigations in patients and family physicians, the likelihood of positive outcomes for patients was linked to the patients’ literacy in modern technologies [31,32]. However, family physicians in our study underscored readily available specialist support and the family practice as a familiar environment for the patient as crucial determinants of clinical benefits. Finally, participants in our study rarely addressed health system factors. This observation is in accordance with other research on barriers for implementation, namely, that health system factors seem to be outside the perception of health care providers [33].

### Strengths and Limitations

Our study has some limitations. First, because of the qualitative nature of our study, family physicians’ anticipated barriers and facilitators of mental health specialist video consultations may not be generalizable to a larger population of family physicians or other health care providers. Thus, our findings may be biased in favor of participants willing to implement video consultations. However, a qualitative approach is most suitable to arrive at an in-depth exploration of the family physicians’ perceptions, and controversial aspects of video consultations were consistently brought up in our study. Second, we used the TICD framework as an analytical concept and applied its key domains to the data. Nevertheless, the original TICD framework subdomains seemed unsuitable for covering the data. Consequently, we decided to generate the subdomains with a bottom-up approach by processing the entire text material available. We think that in doing so, we have further fostered the validity of the final code system. To limit the selection and confirmation bias for the codes generated bottom-up, we reviewed the preliminary code system along the entire material and modified the subdomains when needed. Third, we provide preliminary recommendations for facilitating mental health specialist video consultations in routine primary care derived from the data as a first practical guidance for initial feasibility studies in telepsychiatry for integrated care. However, these recommendations have not yet been evaluated in practice and are therefore not yet evidence based. Finally, none of the participants had any previous practical experience with mental health specialist video consultations. This finding may imply that the anticipated determinants of implementation differ from those that family physicians would mention after having conducted mental health specialist video consultations. Nevertheless, we supported participants in being able to fully visualize the implementation and practice of mental health specialist video consultations by introducing the care model in a video clip (focus groups) or verbal description (telephonic interviews).

### Conclusions

In conclusion, our study points to the precarious situation for patients and family physicians addressing mental health conditions in current everyday practice. Our findings suggest that mental health specialist video consultations show great potential to address the perceived challenges. Given the potential benefits outlined in our qualitative results, we have now embarked on a feasibility study (trial registration number: DRKS00015812) testing a tailored mental health specialist video consultation model for integration into primary care [34]. Specifically, the results presented here have informed the feasibility study protocol and intervention. From the family physicians’ perspective, mental health specialist video consultations hold promise for the future by potentially increasing access to and coordination of specialized care, encouraging cross-sectoral collaboration and providing benefits for patients and family physicians alike.

## Appendix

Multimedia Appendix 1 Semistructured guide for focus groups and telephone interview.

Multimedia Appendix 2 Summary of domains and subdomains.

Multimedia Appendix 3 Recommendations for facilitating mental health specialist video consultations in routine primary care.

## Abbreviations

TICD: Tailored Implementation in Chronic Diseases
