# Supplementary material for: Potential for Integrating Mental Health Specialist Video Consultations in Office-Based Routine Primary Care: Cross-Sectional Qualitative Study Among Family Physicians
Source: J Med Internet Res. 2019 Aug 19;21(8):e13382. doi: 10.2196/13382 (PMC6718083; doi:10.2196/13382)
Supplement: Multimedia Appendix 2 [file jmir_v21i8e13382_app2.docx]

**Multimedia Appendix 2**

**Summary of domains and subdomains**

| *Key domain* | *Definition* | *Subdomains* | *Supporting quotes* |
| --- | --- | --- | --- |
| Individual health professional factors | Factors regarding attitudes and beliefs about the mental health specialist video consultations model related to the individual family physician | Outcome expectancy/potential benefit  (eg, help for patients, saving resources for family physicians) | *Question: “What would be the most attractive factor for you to try out mental health specialist video consultations?”*  *“I could offer something to my patients, not for the purpose of advertisement, but I could actually propose something that makes patients feel better. And that’s why I attend to the patient in the first place.”*  *[Focus Group 4]* |
|  |  | Barriers  (eg, organizational, nonverbal communication) | *“Well, as I said, I think they [mental health specialist video consultations] may work with some people, but in principle it is different from sitting across from someone. There you get information you won’t get through a screen.”*  *[Focus Group 2]* |
| Patient factors | Factors related to patients who might most likely benefit or not benefit from the model as well as factors promoting or inhibiting patient involvement in mental health specialist video consultations | Target groups (eg, the elderly) | *„There are people with impaired personality functioning and mental health conditions who do not manage [to see a mental health specialist]. For those, the low-threshold access [through mental health specialist video consultations] is very important. Otherwise, they just get lost in the system.”*  *[Focus Group 3]* |
|  |  | Patients’ preferences (eg, environmental conditions) | *“If the patient wants to terminate the mental health specialist video consultations saying ‚I can’t continue anymore‘, but is not capable of switching off the device – in such cases someone has to be there.”*  *[Interview 1]* |
|  |  | Barriers (eg, reservations to virtual consultations) | *“If the patient says ‘No, I won’t go for that [mental health specialist video consultations] at all. I won’t even give it a try. I won’t sit myself in front of a box.” – these may be stereotypes I would have to tackle.”*  *[Interview 2]* |
| Professional interactions | Factors related to the cooperation and networking between family physicians and mental health specialists. | Perceptions of responsibilities (eg, medical assistants responsible for administration) | *“I consider it as my genuine task to propose mental health specialist video consultations to patients. Scheduling the appointments will then be done by the girls [medical assistants].”*  *[Interview 2]* |
|  |  | Collaboration (eg, brief case discussions) | *“I think that they [mental health specialist video consultations] support you in your work as a doctor. Afterwards they can be discussed with respect to what has been done, whether there is something urgent to manage and whether something has to be further clarified.”*  *[Focus Group 2]* |
| *Key domains* | *Definition* | *Subdomains* | *Citation* |
| Incentives and resources | (Environmental) factors such as the availability of resources that promote the integration of mental health specialist video consultations into primary care practice routines | Organizational resources (eg, room-availability) | *„There is work to do in the practice beyond the consultation hours. And that is exactly the time, when a room is available, you do not have to rush around and when there is a perfect time window to do something like this [mental health specialist video consultations]. That’s when things calm down in the practice. There is no noise or shuffling of chairs in the waiting room. There is no noise from phone calls at the reception.*  *[Focus Group 2]* |
|  |  | Financial incentives (eg, for the use of a room) | *„Clearly, if it is done in the practice, one could assume that of course the family physician gets something in return.”*  *[Interview 1]* |
| Capacity for organizational change | System and context factors as well as readiness for change related to the primary care setting | none (due to a small number of statements related to this domain) | *Question: “From your point of view, are family physicians currently willing to employ the proposed treatment model?”*  *„It has something to do with being curious. And I can imagine that younger colleagues may be even more curious.”*  *[Focus Group 2]* |
| Social, political and legal factors | Factors regarding social, political and legal prerequisites that may promote or hinder the integration of mental health specialist video consultations into primary care practice routines | none (due to a small number of statements related to this domain) | *„You have to consider that it had been prohibited until recently, the diagnostic assessment by remote consultations.“*  *[Focus Group 1]* |
| Guideline factors | The extent to which the mental health specialist video consultations model may be in line or conflicting with clinical practice guidelines | n/a | n/a |
