# Supplementary material for: Potential for Integrating Mental Health Specialist Video Consultations in Office-Based Routine Primary Care: Cross-Sectional Qualitative Study Among Family Physicians
Source: J Med Internet Res. 2019 Aug 19;21(8):e13382. doi: 10.2196/13382 (PMC6718083; doi:10.2196/13382)
Supplement: Multimedia Appendix 3 [file jmir_v21i8e13382_app3.docx]

**Multimedia Appendix 3**

**Recommendations for facilitating mental health specialist video consultations in routine primary care**

**For buy-in from the primary care practice:**

Family physicians should be won for the idea that mental health specialist video consultations can lead to less fragmented and *more patient-centred care*. The latter may result from increasing access to specialists and fostering collaboration between primary and secondary care.

Applying mental health specialist video consultations and allocating adequate treatment to patients timely may produce some *psychological relief for family physicians* who carry the main responsibility for patients. Family physicians should be made aware of this aspect.

*Benefits concerning patient outcomes, efficiency for processes of care and practice workflows* should be explained to the practice staff.

- Setup and implementation of mental health specialist video consultations should account for existing structures and workflows in the given practice. *Spatial, personnel and time resources used for implementation* should be coordinated with the tightly organized day-to-day routine.
- Medical assistants should be responsible for *administrative and organizational tasks* evolving around the consultations (e.g., appointment allocation and follow-up calls).
- Practice staff and mental health specialists should receive an *initial training* covering the care model itself and the handling of the technology.

**For involving patients in consultations:**

Establishing and monitoring of a *trusted and stable patient-provider-relationship,* that is, between patients and Family physicians as well as patients and mental health specialists, should be a top priority.

- mental health specialist video consultations should *primarily be offered to patients* a) with physical-mental health comorbidity or medically unexplained symptoms, b) hesitating to seek mental healthcare, e.g. due to stigma, and c) immobile patients, particularly in rural areas.
- Family physicians should explicitly *encourage patients* to try out video consultations.

Providers should check all patients potentially benefiting from mental health specialist video consultations for *openness* and *familiarity with computer technology* and virtual communication.

**For the setup and conduct of the consultations:**

- Each patient should continuously consult with the *same mental health specialist*.

Consultations should take place in a *designated room* ensuring confidentiality.

- Practices should consider offering mental health specialist video consultations *outside the usual consultation hours* so that a designated room can be guaranteed. *Fixed time slots* should be used.
- Several *basic requirements* should be met: stable network connectivity, high visual definition, minimized speech delay and instant technical service.

Technical platform and software for consultations should optimally support *nonverbal communication* (e.g., facial expressions and gestures).

The first session should start with a *“warm hand-off”* of the patient from the FP to the mental health specialist. Medical assistants should initialize the subsequent sessions.

A *viable emergency plan* both for patients in severe crises and for technical failures should be put in place. It should clearly delineating responsibilities and tasks for both family physicians and mental health specialists.

**For the collaboration between family physicians and mental health specialists:**

- mental health specialist video consultations can support Family physicians in their attempt for deepening the *collaboration with mental health specialists*.
- Collaboration around mental health specialist video consultations should start based on an *initial kick-off meeting to meet each other in person* and built trust.
- Family physicians should be *responsible for referring patients* to the consultations.
- *Brief case discussions* with specialists via colleague-to-colleague video calls can enable Family physicians to validate diagnostic assessment and initiate tailored treatments.
